# A Case of Neuroma on the Optic Nerve

**Published:** 1864-04

**Authors:** 


A Case of Neuroma of the Optic Nerve, with Remarks and Illustrations. By John A. Lidell, M.D., Surg. U.S. Vol., Prof. of Anatomy in the National Medical College. Second Edition. New York, 1863.

This pamphlet of fifteen pages contains an account of a very interesting case of neuroma of the optic nerve; a tumor which had so developed itself as to crowd the eye forward and downward until it was destroyed, and the tumor occupied the whole orbit. Such cases are very rare, and still more rare that they are reported by so able an anatomist as Dr. Lidell.

